# Mutation of *EMG1* causing Bowen–Conradi syndrome results in reduced cell proliferation rates concomitant with G2/M arrest and 18S rRNA processing delay

**DOI:** 10.1016/j.bbacli.2014.05.002

**Published:** 2014-05-29

**Authors:** Joy Armistead, Richard Hemming, Nehal Patel, Barbara Triggs-Raine

**Affiliations:** a Department of Biochemistry and Medical Genetics, University of Manitoba, Winnipeg, Manitoba, Canada; b Manitoba Institute of Child Health, Winnipeg, Manitoba, Canada

**Keywords:** BCS, Bowen–Conradi syndrome, EMG1, Essential for Mitotic Growth 1, snoRNA, small nucleolar RNA, SAM, S-adenosylmethionine, m1acp3-ψ, N1-methyl-N3-(3-amino-3-carboxypropyl) pseudouridine, CPM, counts per minute, IRES, internal ribosomal entry site., Ribosome biogenesis, Ribosomopathy, Bowen–Conradi syndrome, rRNA, Cell proliferation, EMG1

## Abstract

Bowen–Conradi syndrome (BCS) is a lethal autosomal recessive disorder caused by a D86G substitution in the protein, Essential for Mitotic Growth 1 (EMG1). EMG1 is essential for 18S rRNA maturation and 40S ribosome biogenesis in yeast, but no studies of its role in ribosome biogenesis have been done in mammals. To assess the effect of the EMG1 mutation on cell growth and ribosomal biogenesis in humans, we employed BCS patient cells. The D86G substitution did not interfere with EMG1 nucleolar localization. In BCS patient lymphoblasts, cells accumulated in G2/M, resulting in reduced proliferation rates; however, patient fibroblasts showed normal proliferation. The rate of 18S rRNA processing was consistently delayed in patient cells, although this did not lead to a difference in the levels of 40S ribosomes, or a change in protein synthesis rates. These results demonstrate that as in yeast, EMG1 in mammals has a role in ribosome biogenesis. The obvious phenotype in lymphoblasts compared to fibroblasts suggests a greater need for EMG1 in rapidly dividing cells. Tissue-specific effects have been seen in other ribosomal biogenesis disorders, and it seems likely that the impact of EMG1 deficiency would be larger in the rapidly proliferating cells of the developing embryo.

## Highlights

• EMG1 in healthy and BCS cells co-localizes with ribosome biogenesis factors.
• Cell proliferation rate is reduced in BCS cells.
• BCS cells accumulate at G2/M.
• 18S rRNA biogenesis is delayed in BCS cells.
• We confirm that BCS is a ribosomopathy.

## 1 Introduction

Bowen–Conradi syndrome (BCS, MIM #211180) is a lethal autosomal recessive disorder common in the Hutterite population of the Canadian Prairies and the United States Great Plains. Infants with BCS display central nervous system defects including mental retardation and profound psychomotor retardation. Common physical manifestations include pre- and postnatal growth retardation, microcephaly, prominent nose, micrognathia, rockerbottom feet and flexion contractures of the joints. The average age at death is 13 months, but the exact cause is unclear and seems to be a result of severe failure to thrive. With a prevalence of 1/355 live births, this disease is a significant concern for the Hutterites [1], [2], [3], [4], but has not yet been found outside this population. We identified a single base mutation in *EMG1* (Essential for Mitotic Growth 1) that results in an Asp86Gly (D86G) substitution in the EMG1 protein as the cause of BCS, and showed that this mutation results in an unstable protein, as evidenced by a substantial reduction of EMG1 in BCS patient fibroblasts and lymphoblasts [5], [6]. Little is understood about the role of EMG1 in mammalian cells; however research conducted in yeast has shown that this protein is essential for cell survival and growth, and plays a role in the assembly of the small subunit of the ribosome [7], [8].

Ribosome synthesis and assembly is an extremely complex and energy-intensive cellular process. In fact, in proliferating yeast cells, roughly half of all RNA polymerase II transcription initiation events are on ribosomal protein genes, and approximately 80% of the total RNA in the cell is ribosomal [9]. A mature eukaryotic ribosome is composed of the small subunit, containing the 18S rRNA and 33 ribosomal proteins, and the large subunit, containing the 28S, 5.8S and 5S rRNAs and 49 ribosomal proteins. The 18S, 5.8S and 28S rRNAs are all expressed as a single polycistronic 45S precursor transcript which is processed to yield the mature forms, while the 5S rRNA is transcribed separately. Biogenesis of a fully functioning ribosome involves the coordinated efforts of all three RNA polymerases, and roughly 200 assembly proteins and 75 small nucleolar RNAs (snoRNAs) which aid in the maturation and assembly process [10], [11], [12]. These factors modify nascent rRNA concurrent with transcription, including cleavage of the precursor transcript by endo- and exonucleases, 2′-O ribose methylation, pseudouridylation, base methylation, and assembly with ribosomal proteins. These modifications are thought to optimize rRNA folding, stability and function. Understandably, ribosome biogenesis is tightly regulated, and the TP53 pathway is a powerful checkpoint for ribosome assembly. In a healthy cell, the E3-ubiquitin ligase MDM2 binds and ubiquitylates TP53, targeting it for degradation to maintain low levels of the protein. In the event of aberrant ribosome biogenesis, free ribosomal proteins which are unable to assemble into mature ribosomes bind and segregate MDM2, leading to the accumulation of TP53 [13], [14], [15], [16]. Subsequent cell-cycle arrest or apoptosis ensures that only cells with intact ribosome biogenesis machinery can proliferate.

In yeast, the precursor 45S rRNA is modified co-transcriptionally by the small subunit processome, a complex rivaling the small subunit itself in size, which contains the U3 snoRNA and at least 40 proteins [17], [18]. Emg1, the yeast homologue of the protein mutated in BCS, is associated with the small subunit processome [17] and catalyzes the transfer of a methyl group from a S-adenosylmethionine (SAM) donor to the N1 of a pseudouridine at position 1191 of yeast 18S rRNA (1248 in human 18S rRNA), forming the hyper-modified N1-methyl-N3-(3-amino-3-carboxypropyl) pseudouridine (m1acp3-ψ) [19], [20]. Other roles for Emg1 during ribosome biogenesis have been proposed, including recruitment of the ribosomal small subunit protein RPS19 to the maturing ribosome, and removal of snR57, the snoRNA component of the snoRNP responsible for the 2′-OH ribose methylation of G1570 in yeast 18S rRNA [21]. Most recently, it was posited that Emg1 may act as a chaperone in 18S rRNA folding [22]. As *Saccharomyces cerevisiae* and human EMG1 are 51% identical at the protein level, and human EMG1 complements yeast Emg1 deficiency, it is probable that they have similar functions [8].

Most small subunit processome proteins are essential for cell survival [17], [18], [23], and depletion causes specific ribosome biogenesis deficiencies. In yeast, depletion of Emg1 causes defects in the production of 18S rRNA [17], and processing of precursor rRNA to mature 18S rRNA is delayed in cells lacking the hypermodification of pseudouridine m1acp3-ψ [24]. In addition, cells incorporating the equivalent BCS-causing mutation in Emg1 show reduced growth rates [20]. Assuming similar functions for yeast and human EMG1, we hypothesized that the reduction in EMG1 protein levels seen in BCS patients would result in altered 18S rRNA processing, and therefore deficient biogenesis of the small ribosomal subunit. The reduction of healthy ribosomes would lead to reduced protein synthesis rates concomitant with slower rates of cell proliferation. Because the levels of ribosome proteins are tightly regulated and linked to rRNA transcription to ensure optimal ribosome biogenesis rates, we expected that disruption in ribosome protein levels would also lead to TP53 stabilization. A delay in rRNA processing could consequently be sufficient to disturb proper ribosome biogenesis during periods of increased cell proliferation, such as embryonic development. Similar defects have been found in the ribosomopathies in humans, a clinically variable group of disorders which are associated with mutations in ribosome proteins and biogenesis factors (reviewed in [25], [26], [27]). Here, we show that cell proliferation rates were significantly reduced in patient lymphoblasts concomitant with a cell cycle delay at G2/M, without detectably altering steady-state levels of the small subunit of the ribosome, 18S rRNA levels, or protein synthesis rates. In addition, processing of 18S rRNA was slightly, but consistently, delayed in both lymphoblasts and fibroblasts from BCS patients.

## 2 Materials and methods

### 2.1 Study subjects

Lymphoblast or fibroblast cell lines from patients were generated as part of a previous study to map the BCS gene [3]. Signed informed consent was obtained for all participants. This study was approved by the Health Research Ethics Board at the University of Manitoba. Unless otherwise stated, all experiments were performed in both lymphoblasts and fibroblasts. For each cell type, two patient cell lines were compared with two to three age-matched control cell lines.

### 2.2 Cell culture

Fibroblast cells were maintained in minimal essential medium (alpha modification) (Invitrogen, Sigma) with 10% fetal bovine serum and lymphoblasts were maintained in RPMI medium 1640 (Invitrogen, Sigma) with 15% fetal bovine serum, at 37 °C and 5% CO_2_. All media contained 100 U penicillin/ml and 100 μg streptomycin/ml (Invitrogen).

### 2.3 Immunocytochemistry

Cells were grown on glass coverslips, were rinsed with phosphate buffered saline (PBS) (1.9 mM NaH_2_PO_4_, 8.1 mM Na_2_HPO_4_, 150 mM NaCl, pH 7.4), and were fixed in 4% paraformaldehyde for 15 min at room temperature. After three washes in PBS, quenching was performed in 50 mM ammonium chloride in PBS for 10 min, followed by another three washes in PBS. Permeabilization was done with 0.1% Triton X-100 in PBS for 10 min, the coverslips were washed three times in PBS, and the cells were incubated in 100 μl blocking solution (3% bovine serum albumen, 0.05% Tween in PBS) for 60 min. Primary antibodies (100 μl) were diluted in blocking solution as follows: EMG1 rabbit polyclonal at 1:500 (Protein Tech Group), NOP14 mouse polyclonal at 1:100 (Novus), fibrillarin mouse monoclonal at 1:500 (Abcam) and incubated for 1 h. Incubation with secondary antibodies diluted at 1:1000 (Alexa Fluor, Molecular Probes) was performed for 30 min. The coverslips were rinsed twice with water, and incubated for 30 s in Hoechst 33342 diluted 1 μl in 100 ml of water to stain nuclei. The coverslips were washed twice in water, Prolong Gold Antifade Reagent (Molecular Probes) was applied to a glass slide, and the coverslip was inverted on top, cured overnight, and sealed with clear nail polish.

### 2.4 Assessment of cell proliferation rate

Cell proliferation rates were determined for each cell line using an MTT assay kit (Cell Titer 96 Non-Radioactive Cell Proliferation Assay kit, Promega) following the manufacturer's instructions. Briefly, 1 × 10^4^ cells were seeded into the wells of 24-well plates and grown at 37 °C for 0, 24, 48 or 72 h. At each time point, the cells were incubated at 37 °C for 4 h in a 150 μl MTT reagent, followed by 1 h in 1 ml of stop solution. The absorbance of each well was read by a spectrophotometer at 570 nm. Alternatively, cells were counted manually at twelve hour intervals using a hemacytometer. Dead cells were excluded using trypan blue.

### 2.5 Cell cycle analysis

Cells were harvested and washed twice with wash buffer (0.1% fetal bovine serum in PBS) to derive a single cell suspension. Equal numbers of cells were suspended in 1 ml of buffer, and 3 ml of − 20 °C absolute ethanol were added dropwise with light vortexing to fix cells while minimizing clumping. Cells were fixed a minimum of 3 h at 4 °C and stored at room temperature until the day of analysis. Fixed suspensions were washed twice with PBS, suspended in 1 ml of staining solution (40 μg/ml propidium iodide, 0.2 ml RNase A, 0.1% Triton X-100 in PBS), and incubated at room temperature for 30 min. Samples were placed on ice until analysis by flow cytometry, using a MoFlo XDP cell sorter (Beckman Coulter) and Modfit LT software for cell cycle analysis (Verity Software House).

### 2.6 Detection of ribosomal subunits

Ribosomal subunits were detected using a modified method [28]. Cells in log phase of growth were washed with PBS and pelleted by centrifugation. Equal numbers of cells (2 × 10^6^ for fibroblasts and 1.7 × 10^7^ for lymphoblasts) were lysed in ribosome extraction buffer (10 mM Tris pH 7.4, 1% Igepal, 0.5% sodium deoxycholate, 2 mM [low] or 15 mM [high] MgCl_2_, 0.1 M KCl, 0.5 mg/ml Heparin, 80 U/ml RNAseOut [Invitrogen], 1 mM DTT, 1/250 protease inhibitor stock [Sigma]) to extract the ribosomes. After a 15 minute incubation on ice, nuclei and cell debris were pelleted by centrifugation at 12 000 ×*g* for 2 min. A portion of the lysate was saved as an input fraction. Approximately 300 μl of the ribosome lysate was applied to the top of a 10–45% sucrose gradient in sucrose gradient buffer (Tris pH 7.4, 0.1 M KCl, MgCl_2_ equal to ribosome extraction buffer, 2 mM EDTA [low Mg gradients only]) prepared in polyallomer ultracentrifuge tubes. Gradients were separated by centrifugation at 36 000 rpm for 3 h at 4 °C with the brake off. Gradients were collected with an ISCO gradient collector by pumping 60% sucrose in the bottom while the gradient was collected from the top and absorbance was monitored at 254 nm, and 0.5 ml fractions were collected. The tracing produced was scanned, and the area under the curve was determined using the Measure function in Axiovision software. Average 60S/40S ratios for wild type and BCS cells were compared using Student's *t*-test.

### 2.7 Immunoblot analysis

Proteins were isolated from cultured cells by lysis in a modified radioimmunoprecipitation assay (RIPA) buffer (1% tert-octylphenoxy (poly) oxyethelene ethanol [IGEPAL]; 0.5% sodium deoxycholate; 0.1% SDS; 1.9 mM NaH_2_PO_4_; 8.1 mM Na_2_HPO_4_; 150 mM NaCl), supplemented 1 in 500 with a protease inhibitor cocktail (Sigma). RIPA-insoluble proteins were separated by centrifugation for 10 min in a microcentrifuge at 10 000 ×*g*, leaving the RIPA-soluble proteins in the supernatant. Protein concentrations were determined using either the Bradford assay (Bio-Rad) or the bicinchoninic acid assay (Thermo Scientific). Equal amounts of protein (20–25 μg) were separated at 160 V using 10% SDS-PAGE gels. Proteins were transferred to a nitrocellulose membrane in N-cyclohexyl-3-aminopropanesulfonic acid (CAPS) buffer at pH 11 (0.01 M CAPS, 10% methanol) or in Tris–glycine transfer buffer (25 mM Tris, 192 mM glycine, 20% methanol) at 100 V for 1 h according to a modified method [29]. The membrane was blocked in 5% skim milk in Tris buffered saline Tween (TBST) (20 mM Tris pH 7.4; 0.15 M NaCl; 0.1% Tween) for 1 h. Individual proteins were detected using a primary antibody diluted in blocking solution overnight at 4 °C. The membrane was washed three times with TBST, incubated with horseradish peroxidase (HRP)-conjugated secondary antibody for 1 h, washed, and visualized using the Immobilon western chemiluminescent HRP substrate (Millipore). Primary antibody dilutions were used as follows: RPL7 rabbit polyclonal at 1:5000 (Bethyl laboratories), RPS19 rabbit polyclonal at 1:3000 (Proteintech Group), TP53 2B2.68 mouse monoclonal at 1:2000 (Santa Cruz), p21/WAF1/Cip1 mouse monoclonal at 1:250 (Millipore), and β-actin mouse monoclonal at 1:5000 (Sigma). The secondary donkey anti-rabbit and donkey anti-mouse antibodies (Jackson Immunoresearch) were diluted to 1:20 000.

### 2.8 Isolation of total RNA and gel electrophoresis

Total RNA was isolated from fibroblasts or lymphoblasts using the TRIzol reagent (Invitrogen) and following manufacturer's instructions. Cells were lysed in the culture vessel by the addition of TRIzol and pipetting up and down multiple times. Cells grown in suspension were pelleted and the supernatant removed before lysis. Chloroform (0.2 ml) was added to lysates and shaken by hand for 15 s to mix, then incubated at room temperature for 3 min. Samples were centrifuged at 12 000 ×*g* 15 min at 4 °C, and the upper aqueous phase was transferred to a new tube. RNA was precipitated in 0.5 ml isopropanol, and rotated for 10 min at room temperature. After centrifugation, the supernatant was decanted, and the RNA pellet was washed with 1 ml 75% ethanol. The ethanol was removed, the pellet was air dried, and the RNA was resuspended in diethylpyrocarbonate-treated water and stored at − 80 °C. To prepare RNA for separation by gel electrophoresis, equal volumes of RNA and NorthernMax-Gly Sample Loading Dye (Ambion) were mixed and incubated at 50 °C for 30 min to eliminate secondary structures. RNA samples were then separated by gel electrophoresis in a 1% agarose BPTE gel (100 mM PIPES, 300 mM Bis-Tris, 10 mM EDTA pH 8.0) in 1 × BPTE buffer at 5 V per cm.

### 2.9 Determination of 28S/18S rRNA ratio

For each cell line, three independent samples of RNA were analyzed by capillary electrophoresis using the RNA 6000 Nano LabChip Kit in the Agilent 2100 Bioanalyzer according to the manufacturer's instructions. Samples (1 μl) were analyzed in duplicate compared to 1 μl of ladder. The quality of RNA separation was assessed by the resolution of the individual peaks in the ladder, and the area of each peak, as well as the 28S/18S rRNA ratios, were determined using the Agilent Bioanalyzer software.

### 2.10 Metabolic labeling and analysis of ribosomal RNA

Pulse-chase metabolic labeling of rRNA was conducted essentially as described by Pestov et al. [28]. ^32^P-orthophosphate labeling was employed in both fibroblast and lymphoblast cell lines, and repeated using ^3^H methyl-methionine in fibroblasts. For ^32^P-orthophosphate labeling, equal numbers of fibroblasts (~ 2.5 × 10^5^ cells per well) were cultured overnight in six-well plates in DMEM (Invitrogen). The following morning, cells were starved for 1 h in phosphate-free DMEM (Sigma) supplemented with dialyzed serum, then 10 μCi/ml ^32^P-orthophosphate (Perkin Elmer) was added for a pulse period of 40 min. Cells were washed twice in complete DMEM and incubated in cold medium. Cells were harvested at 20 minute intervals using the TRIzol reagent (Invitrogen), and RNA was isolated as described above. Incorporated radioactivity was determined in counts per minute (CPM) using a scintillation counter (Beckman Coulter), and equal counts from each sample were mixed with glyoxal loading dye (Ambion). RNA species were separated by gel electrophoresis as above. The gel was dried and exposed to a phosphor storage screen (Bio-Rad) which was subsequently scanned using a phosphorimager (Bio-Rad). Band intensities were quantified using Image Lab software (Bio-Rad).

For ^3^H methyl-methionine labeling, fibroblasts were treated essentially as for ^32^P-orthophosphate labeling, except that cells were starved for 30 min in cysteine/methionine-free DMEM (Invitrogen) supplemented with 5 mM cysteine, dialyzed serum, and glutamine. Cells were labeled with 50 to 75 μCi/ml l-[methyl-^3^H] methionine (Perkin Elmer) for 30 min, then chased in complete DMEM supplemented with 5 mM methionine for the indicated time periods. Following gel electrophoresis, RNA was transferred to a positively charged nylon membrane (GE Healthcare) in BPTE using the Genie apparatus (Idea Scientific) at 12 V for 1 h at 4 °C. The membrane was then exposed to a phosphor storage screen (Screen K/Tritium, Bio-Rad).

### 2.11 Assessment of protein synthesis rate

Cells were incubated for 4 h at 37 °C with media containing 150 μCi ^35^S Cys/Met (Perkin Elmer), washed with ice-cold PBS, and lysed in 500 μl of radioimmunoprecipitation assay (RIPA) buffer (1% tert-octylphenoxy (poly)oxyethelene ethanol [IGEPAL]; 0.5% sodium deoxycholate; 0.1% SDS; 1.9 mM NaH_2_PO_4_; 8.1 mM Na_2_HPO_4_; 150 mM NaCl) supplemented with protease inhibitors (Sigma). After centrifugation, a 20 μl aliquot of the supernatant was mixed with 100 μl of 40 mg/ml bovine serum albumin and 1 ml of ice-cold 10% trichloroacetic acid (TCA). After incubating on ice for 45 min, the precipitated protein was collected on a 1.2 μm glass microfiber filter (GF/C, Whatman) using a vacuum manifold. The filters were washed twice with 10% TCA, once with 100% ice-cold ethanol, and the filter-bound radioactivity was determined in CPM using a liquid scintillation counter. The protein concentration for each sample was determined using the bicinchoninic acid assay (Pierce). Protein synthesis rate was then calculated as the amount of incorporated radioactivity in CPM per μg of protein. Cycloheximide (Sigma) was used as a negative control to inhibit protein synthesis [30].

### 2.12 Statistical analyses

Statistical significance was assessed using Student's two-tailed *t*-test calculated by GraphPad Prism software. A p value of less than 0.05 was considered statistically significant.

## 3 Results

### 3.1 EMG1 co-localizes with ribosome biogenesis factors

**Fig. 1:**
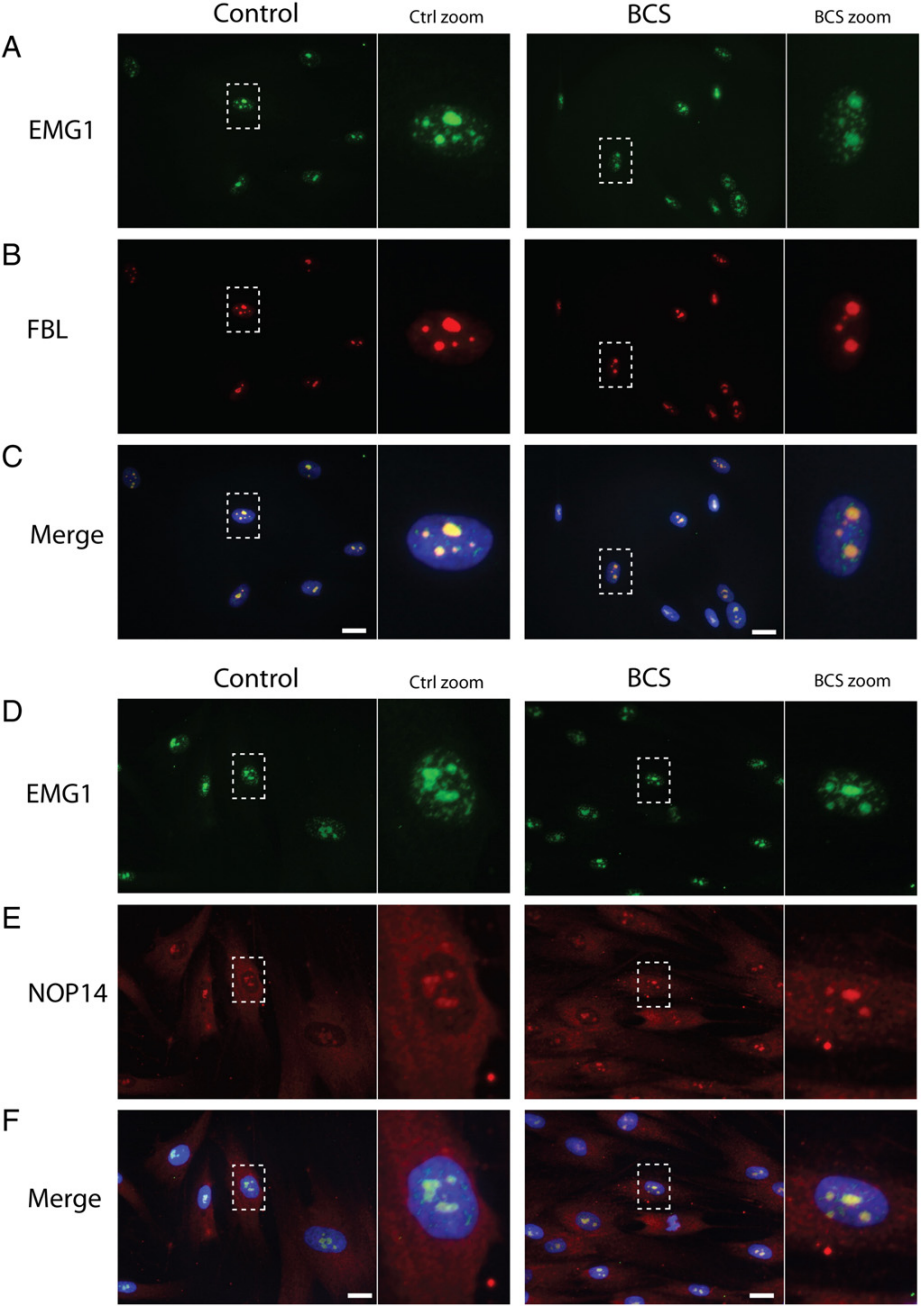
EMG1 co-localizes with ribosome biogenesis factors in nucleoli of human cells. (A) Fibroblasts were fixed using 4% formaldehyde and stained with anti-EMG1 (green) and anti-fibrillarin (red) in control and patient cells. DNA was visualized by staining with Hoechst 33342 (blue). The merged photo shows the co-localization of EMG1 and the dense fibrillar component protein fibrillarin, in nucleoli of both control and BCS cells. Original magnification, 63 ×; scale bar = 20 μm. (B) Fibroblasts were stained with anti-EMG1 (green) and anti-NOP14 (red). The merged photo shows the co-localization of EMG1 and the small subunit processome protein NOP14 in nucleoli. Similar localization was found in normal control and BCS patient cells. Original magnification, 63 ×; scale bar = 20 μm.

EMG1, the protein product of the gene mutated in Bowen–Conradi syndrome, has been implicated in ribosome biogenesis in yeast and human cells, although most work to determine its function has been performed in yeast [7], [8], [19], [20]. To evaluate the sub-cellular localization of EMG1 relative to other ribosome biogenesis factors in human cells, immunofluorescence was used to detect EMG1, fibrillarin, and NOP14. As expected, EMG1 was localized primarily to the nucleolus of both BCS patient and control fibroblasts, as evidenced by its co-localization with fibrillarin, an rRNA methyltransferase found in the dense fibrillar component of the nucleolus [31] (Fig. 1A–C). NOP14, a small ribosomal subunit assembly factor which has been shown to interact with EMG1 [7], was detected in the nucleolus and in the cytoplasm, in agreement with previous analysis of its localization in yeast cells by biochemical fractionation and by immunofluorescence [7] (Fig. 1E). EMG1 largely co-localized with the nucleolar fraction of NOP14 (Fig. 1D–F). EMG1 in lymphoblasts displayed a similar nucleolar localization (not shown). These results support a role for EMG1 in ribosome biogenesis, and, importantly, indicate that EMG1 localization is unaffected in BCS patient cells.

### 3.2 Cell proliferation and cell cycle are delayed in BCS lymphoblasts but not in fibroblasts

**Fig. 2:**
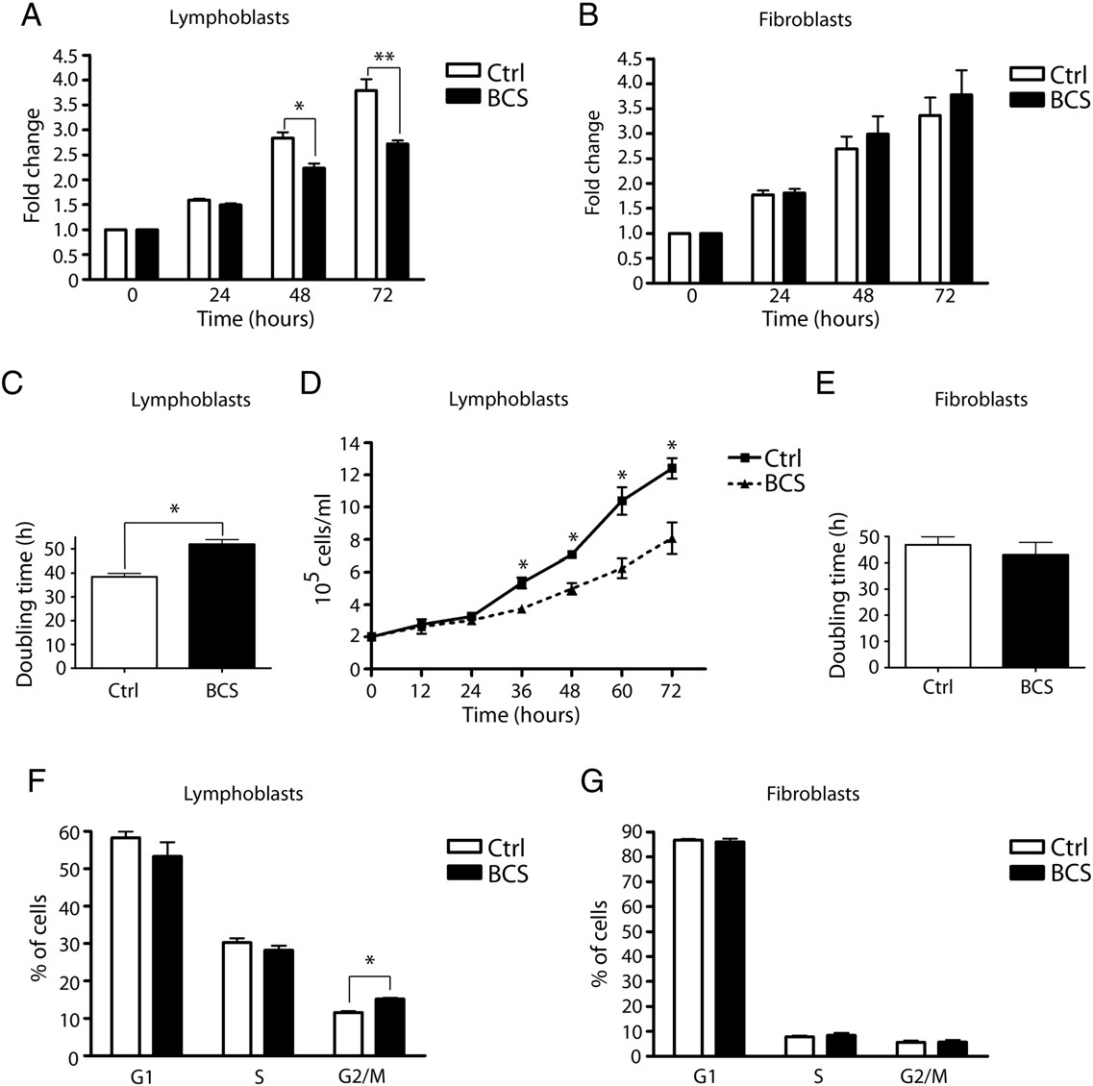
BCS patient lymphoblasts display reduced cell proliferation rates and cell cycle delay at G2/M. (A, B) Cell proliferation rates, evaluated using the MTT assay, in lymphoblasts (A) and fibroblasts (B). At 24-hour intervals, cells seeded in a 96-well plate were incubated with the MTT reagent for 4 h, lysed, and the absorbance was read at 570 nm. The fold change relative to time 0 was plotted for each time point. The mean of four independent experiments, each performed in triplicate, is shown for the lymphoblasts, and three experiments for the fibroblasts. BCS lymphoblast results were significantly lower than controls at 48 h (*p < 0.01), and at 72 h (**p < 0.001); error bars show SEM. (C, E) Doubling time of lymphoblasts (C) and fibroblasts (E). Doubling time was calculated from the results of the MTT assay performed in (A) and (B). In BCS lymphoblasts, doubling time was significantly longer (*p < 0.0001). (D) Lymphoblast cell concentrations in cells per milliliter, counted at twelve hour intervals. BCS lymphoblast numbers were similar to control numbers until the 36 hour time point, and were significantly lower than controls from 36 to 72 h (*p < 0.01). The mean of two independent experiments, each performed in duplicate, and SEM are shown. (F, G) Cell cycle analysis of lymphoblasts (F) and fibroblasts (G). Cells were treated with propidium iodide and sorted based on DNA content. In the lymphoblasts, the percentage of cells in G2/M phase was significantly higher in BCS cells than in control cells (*p = 0.00185). The mean of three independent experiments and SEM are shown.

BCS patients display severe pre- and post-natal growth retardation, and Emg1 is essential for cell growth in yeast [7], [8] and in mice [32]. To determine if growth of BCS patient cells was altered due to the *EMG1* mutation, the MTT assay was employed to compare proliferation rates in BCS patient and age-matched control lymphoblasts or fibroblasts. This assay employs a yellow tetrazolium salt substrate which is reduced to a purple insoluble formazan complex by the mitochondria. The amount of color produced is therefore directly proportional to the number of metabolically active cells. BCS patient lymphoblasts showed significantly slower proliferation rates over a 72-hour period relative to control lymphoblasts (Fig. 2A), with a mean doubling time of 51.90 ± 2.03 h, compared to a mean of 38.40 ± 1.38 h for control lymphoblasts (Fig. 2C). We found no significant difference in the proliferation rates between unaffected control fibroblasts and BCS patient fibroblasts (Fig. 2B). The mean doubling time for control fibroblasts was 46.73 ± 3.10 h, and 43.02 ± 4.68 h in BCS fibroblasts (Fig. 2E). To rule out the possibility that the lymphoblast MTT assay data were due to changes in cell metabolism and not in cell numbers, cells were manually counted at twelve hour intervals, using trypan blue to exclude dead or dying cells (Fig. 2D). BCS lymphoblasts proliferated significantly slower than control cells, confirming the MTT assay results.

It has been shown previously that errors in biogenesis of the small ribosomal subunit cause a stall in the cell cycle at G1/S, due to a checkpoint which assesses adequate ribosome numbers [13], [17], [33]. A lengthening of G1/S phase could explain the slower cell proliferation rate, as cells would take longer to progress through a complete cell cycle. To determine if the cell cycle was altered in BCS, cell sorting of propidium iodide-stained cells was performed. This assay separates cells into G1, S, or G2/M populations based on their DNA content. Unexpectedly, lymphoblasts from BCS patients had a significantly increased proportion of cells in G2/M (Fig. 2F), with no significant difference in any other phase of the cell cycle. On the other hand, cell cycle was not altered in BCS patient fibroblasts when compared to controls (Fig. 2G). These data suggest that a stall at the G2/M checkpoint results in a longer cell cycle in BCS patient lymphoblasts, causing reduced proliferation rates, but that patient fibroblasts are not appreciably affected.

### 3.3 Levels of the small ribosomal subunit are normal at steady state in BCS patient cells

**Fig. 3:**
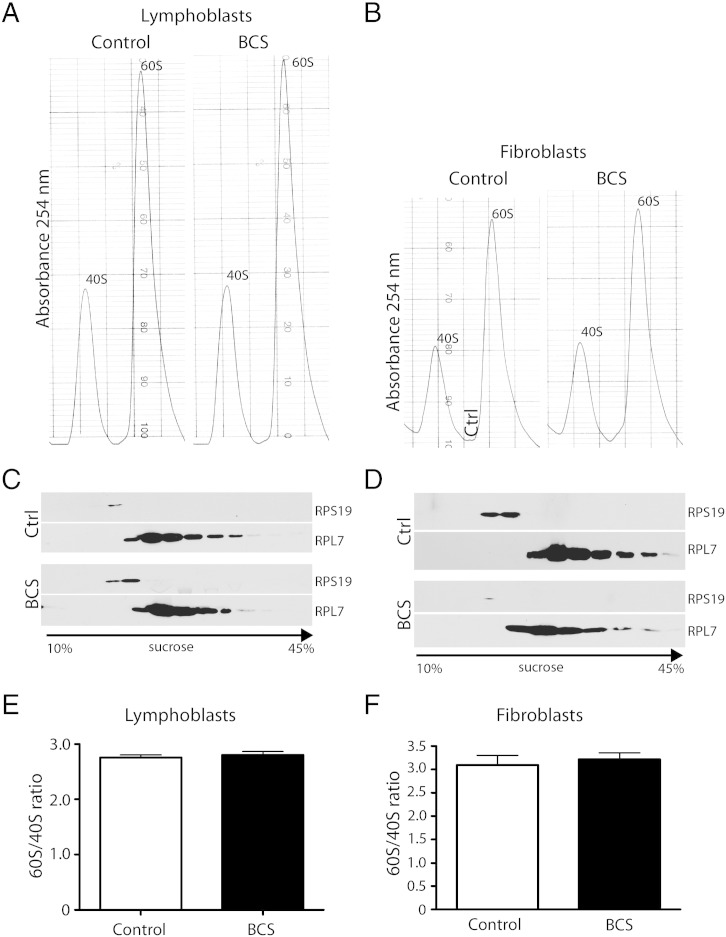
Ribosomal subunit levels at steady-state are normal in BCS patient cells. (A, B) Sucrose gradient separation of 40S and 60S ribosomal subunits in lymphoblasts (A) and fibroblasts (B). Ribosomal subunits were detected following sucrose gradient separation by absorbance at 254 nm, and examples of the resultant tracings are shown. (C, D) Fractions were collected at regular intervals and probed by immunoblot analysis for ribosomal proteins using anti-RPS19 and anti-RPL7 antibodies to verify the separation of the small (RPS) and large (RPL) subunits. (E, F) The area under the curve was determined for individual peaks, and the ratio of large to small subunit was calculated. No significant difference between unaffected control and BCS-affected cells was found in lymphoblasts (E) and fibroblasts (F). The mean of six individual experiments and SEM are shown.

In order to determine if the deficiency of EMG1 protein seen in BCS patient cells was causing an alteration in the synthesis of the small ribosomal subunit, the relative amounts of the small and large ribosomal subunits were evaluated. Lysates prepared from equal numbers of patient and unaffected control lymphoblasts and fibroblasts, were separated in a sucrose gradient and the ribosomal subunits were detected by absorbance as they were collected from the gradient (Fig. 3A, B). Ribosomal proteins of the small and large subunits were monitored by immunoblot in collected fractions to verify separation (Fig. 3C, D). The ratio of the large subunit to the small subunit was compared between BCS patients and controls, and no significant difference was found (Fig. 3E, F), either in the presence of low (Fig. 3A, B) or high (not shown) levels of Mg^2 +^, which is required for the association of the subunits. These data indicate that at steady state, the small subunit of the ribosome is at normal levels in BCS patient cells relative to the large subunit. No difference in total ribosome subunit levels was evident between control and patient cells (data not shown), ruling out the possibility that total ribosome synthesis is down-regulated in BCS patients.

### 3.4 The rate of 18S rRNA processing is delayed in BCS patient cells

**Fig. 4:**
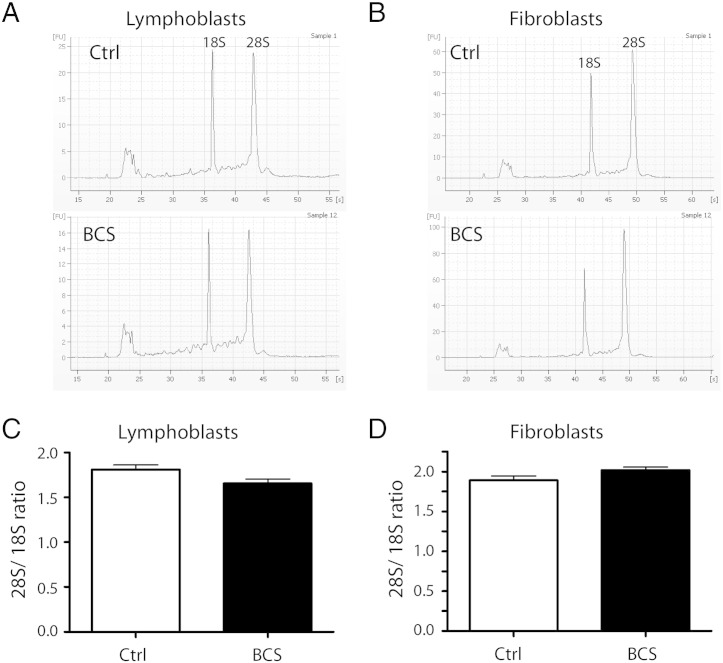
Ribosomal RNA levels at steady-state are normal in BCS patient cells. (A, B) Total RNA was isolated from unaffected control and BCS-affected lymphoblasts (A) and fibroblasts (B), and separated by capillary chromatography using the 6000 RNA Nano kit in an Agilent Bioanalyzer. The resulting electropherograms show the 18S and 28S peaks, as well as a smaller peak which encompasses the 5S, 5.8S, and tRNA. (C, D) 28S/18S ratios for lymphoblasts (C) and fibroblasts (D). The area of each peak was calculated by the Agilent software, and the ratios of 28S to 18S rRNA were calculated. The mean of three individual experiments, performed in triplicate, and SEM are shown. No significant difference was found between the control and BCS patient cells.

The amount of steady-state 18S rRNA in cells from BCS patients was then assessed. Total RNA was isolated from either fibroblasts or lymphoblasts from BCS patients or normal, age-matched controls, and was analyzed using the RNA 6000 Nano LabChip Kit in the Agilent 2100 Bioanalyzer. Examples of the resultant electropherograms are shown in Fig. 4A–B. The area under the curve for each peak was calculated, and total amounts of 18S were evaluated relative to 28S rRNA. In each case, there were no significant differences in the ratio of 28S rRNA to 18S rRNA between any of the samples (Fig. 4C, D).

**Fig. 5:**
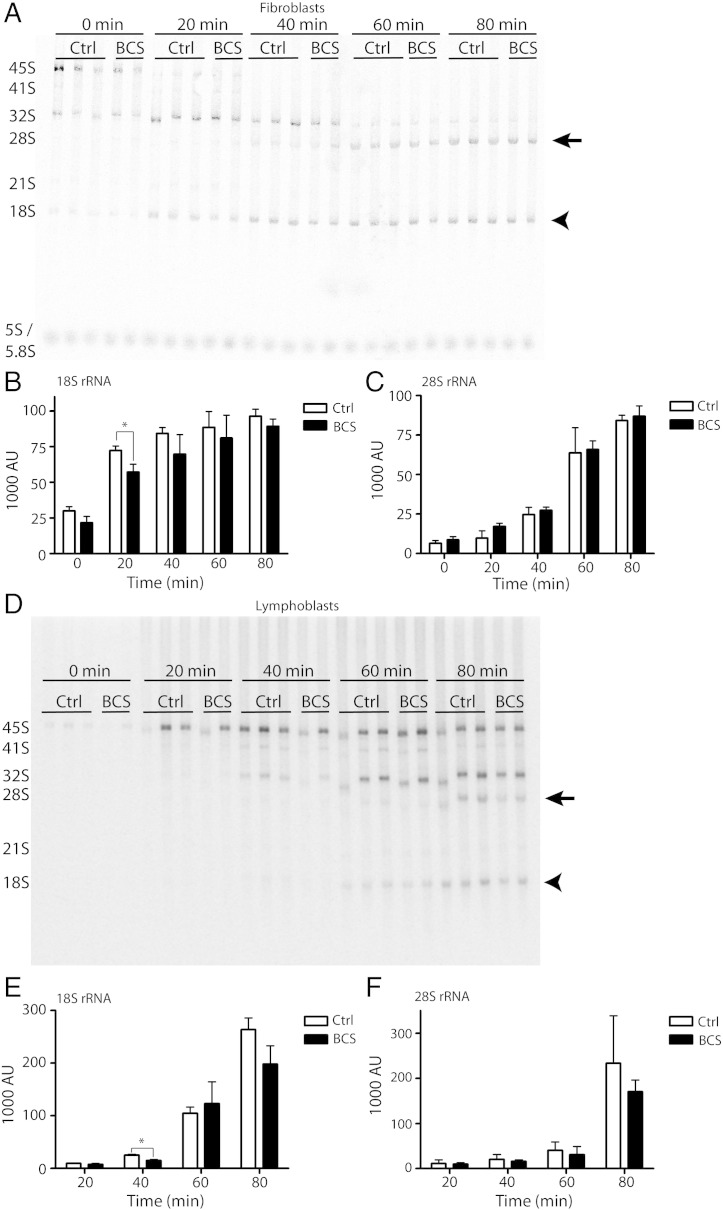
Ribosomal RNA processing is delayed in BCS patient cells. (A) In fibroblasts, nascent rRNA was metabolically labeled using (methyl-^3^H) methionine for 30 min, and RNA was isolated at twenty minute intervals to follow the processing rate of the large 45S rRNA precursor to the mature 18S (arrowhead) and 28S species (arrow). Equal counts were separated on a 0.7% agarose gel, and transferred to a positively-charged nylon membrane. The membrane was exposed to a phosphor storage screen, which was scanned using a phosphorimager. The precursor 45S rRNA, the 28S and 18S mature species, and the intermediates are indicated on the left side of the diagram. A representative image of three independent experiments is shown. (B, C) The intensity of each band in (A) was quantified using Image Lab software. The graphs indicate the mean density in arbitrary units of the 18S (B) and 28S rRNA (C) bands at each time point, and the error bars represent standard deviation. For this experiment, the average of three control cell lines and two BCS cell lines are shown. The 18S rRNA level was significantly reduced in BCS cells at the 20 minute time point (*p = 0.0287). (D) A representative image of an rRNA processing experiment in lymphoblasts. The experiment was performed essentially as in (A), except the rRNA was labeled with ^32^P_i_ for a twenty minute pulse. Following gel electrophoresis to separate the RNA species, the gel was dried and directly exposed to a phosphor storage screen. (E) and (F) show the intensity of each band. The 18S rRNA was significantly reduced in BCS cells at the 40 minute time point (*p = 0.0292).

Although the steady-state levels of rRNA remain unaffected, it is possible that the processing rate of precursor 45S to mature 28S and 18S rRNA is delayed in BCS patient cells. This was previously shown in the ribosomopathy Shwachman–Diamond syndrome, where sucrose gradient analysis showed similar steady-state levels of ribosomal subunits, yet the formation of mature rRNA was significantly slower in Shwachman–Diamond syndrome patient fibroblasts [34]. To evaluate rRNA processing rates, pulse-chase analysis was employed using either ^32^P_i_ or ^3^H-methyl methionine to label nascent rRNA. Using this technique, it is possible to follow the transformation of precursor 45S rRNA to its mature 28S (Fig. 5, arrows) and 18S rRNA forms (arrowhead) over time. Following a pulse of radioactive label, the cells were chased in cold media, and samples were collected at regular intervals. Ribosomal RNA was isolated and separated using gel electrophoresis, then visualized using autoradiography (Fig. 5). Using Image Lab software to quantify band intensity, a consistent reduction in labeling of 18S rRNA in BCS patient cells was found (Fig. 5B and E), while 28S rRNA was unaffected (Fig. 5C and F). Although this difference was only statistically significant at a specific point in processing (see time 20 min of 18S rRNA in fibroblasts, Fig. 4B; and time 40 min of 18S rRNA in lymphoblasts, Fig. 4E), the reduction was consistent through several independent experiments, employing both the ^32^P_i_ and the ^3^H methyl-methionine labels, and in both lymphoblasts and fibroblasts. This, together with the fact that steady state rRNA levels were normal, indicates that overall rRNA production occurs at a normal rate in BCS cells, and that processing of 18S rRNA is instead delayed. This delay does not detectably affect steady-state levels of 18S or 28S rRNA, nor does it alter steady-state levels of the ribosomal subunits.

### 3.5 Protein synthesis rates are unaffected in BCS patient fibroblasts

**Fig. 6:**
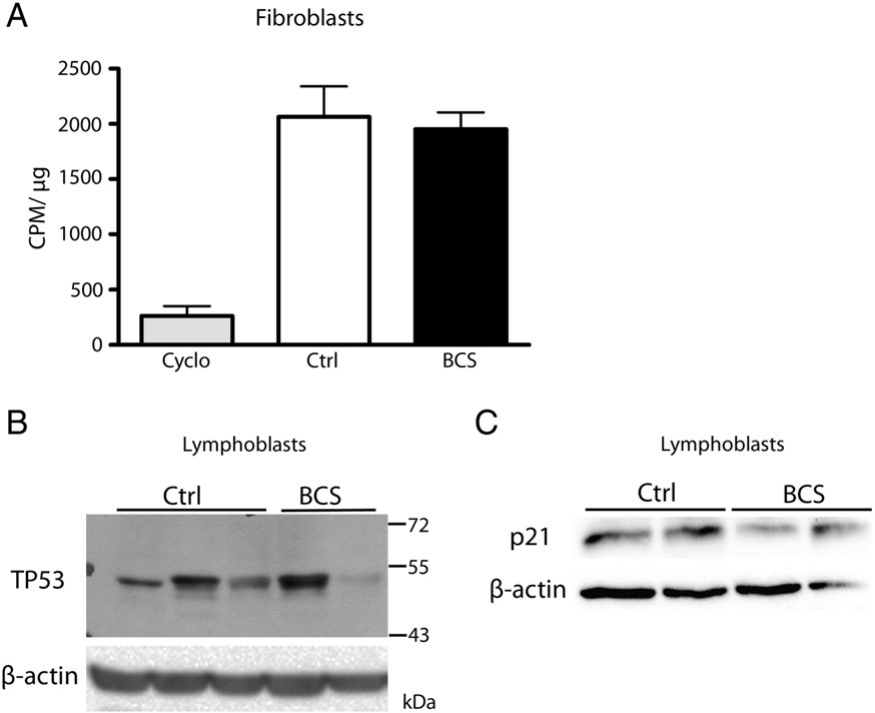
Protein synthesis rates and TP53 levels are unaffected in BCS patient cells. (A) Fibroblasts were incubated with ^35^S-cysteine/methionine to label newly synthesized protein, while cycloheximide was included with control cells to inhibit protein synthesis as a negative control. Proteins were TCA-precipitated from cell lysates, filtered, and washed. Labeled protein was detected using a liquid scintillation counter, protein concentration was determined, and counts per microgram of protein was calculated. No statistical difference was found. The mean of three independent experiments and SEM are shown. (B) Lymphoblast lysates were probed by immunoblot using an anti-TP53 antibody, and β-actin is shown as a loading control. TP53 levels in the BCS cells were variable, and no statistically significant difference was found between control and BCS cell lines. A representative image from three independent experiments is shown. (C) The presence of p21 in lymphoblast lysates was assessed by immunoblot using an anti-p21 antibody, and the same blot was probed for β-actin as a loading control. No significant difference between control and BCS cell lines was found.

A delay in the 18S rRNA processing could result in sub-optimal ribosome function, and perturb overall protein synthesis rates. We therefore examined the rate of protein synthesis in each fibroblast cell line by the incorporation of ^35^S Cys/Met over a four-hour period, calculated as the amount of incorporated radioactivity in CPM per μg of protein (Fig. 6A). Similar results were found for BCS patient and unaffected control fibroblasts, indicating that the delay in 18S rRNA processing does not alter the rate of translation by the ribosome. This is consistent with the conclusions that the delay in 18S rRNA processing is transient, and does not affect steady-state levels of the ribosomal subunits. Given that the ribosomal RNA and subunit levels were similarly unaffected in BCS patient lymphoblasts, the protein synthesis rate experiments were performed on fibroblasts only.

### 3.6 TP53 is not stabilized in BCS patient cells

Because the dysregulation of ribosome biogenesis has been shown to lead to TP53 stabilization and cell cycle arrest [13], [14], [15], [16], levels of TP53 protein in BCS patient cells and unaffected controls were compared using immunoblot analysis. At steady state, TP53 protein levels varied between cell lines, with no clear difference between BCS patient cells and unaffected controls in lymphoblasts (Fig. 6B) or fibroblasts (not shown). To assess downstream targets of TP53, we examined levels of p21 in lymphoblasts using an anti-p21 antibody, and again found no differences between control and patient cell lines (Fig. 6C). The absence of consistent TP53 stabilization or induction of downstream p21, suggests that the cell proliferation defect in BCS lymphoblasts is not mediated by TP53. This is consistent with the fact that an *Emg1* knockout mouse is not rescued by Trp53 ablation [32].

## 4 Discussion

In yeast, a reduction in levels of the ribosome biogenesis enzyme Emg1 causes a decrease in 18S rRNA levels with a corresponding accumulation of its immediate precursor in the rRNA processing pathway [7]. This lack of 18S rRNA leads to an imbalance between the large and small ribosomal subunits, which in turn causes a reduction in protein synthesis and cell proliferation rates [8]. Thus, we expected that since EMG1 levels are severely reduced in BCS patient lymphoblasts and fibroblasts, we would see a corresponding decrease in cell proliferation and protein synthesis rates due to a deficiency of small ribosomal subunits. Instead, we found slower proliferation rates in BCS lymphoblasts, accompanied by an increase in the proportion of cells in G2/M, while neither was affected in BCS fibroblasts. A consistent, transient delay in 18S rRNA processing in both fibroblasts and lymphoblasts was found, although it did not result in a detectable alteration of steady-state levels of 18S rRNA or small ribosomal subunits. Protein synthesis rates were also unaffected in BCS patient fibroblasts.

The reduced proliferation rate, observed only in the BCS lymphoblasts, can be explained by a significant lengthening of the cell cycle in G2/M, which was also found only in lymphoblasts. While rRNA processing defects typically cause G1 arrest [13], [17], [33], an increase in the percentage of cells in G2/M phase has been reported in cells depleted of dyskerin, the protein responsible for the X-linked form of the ribosomopathy dyskeratosis congenita [35], [36]. In addition, changes in ribosomal protein RPL13a expression can induce cell cycle arrest at G2/M [37], mutations in RNAse MRP causing cartilage hair hypoplasia lead to cell cycle delay in anaphase [38], and a mutation in *pescadillo* in zebrafish, a gene implicated in ribosome biogenesis, disrupts oligodendrocyte formation due to failure to progress from S phase to mitosis [39]. The stall at G1/S seen in many cases of ribosome biogenesis disruption has been shown to be the result of a checkpoint which verifies that adequate numbers of ribosomes are present before proceeding with the cell cycle. We found that steady-state levels of the ribosome subunits are normal in BCS cells, which may explain why they are able to progress through the G1/S checkpoint. A second checkpoint may exist preventing cells from progressing through mitosis in the absence of proper ribosome biogenesis.

Intriguingly, in the ribosomopathy X-linked dyskeratosis congenita, ribosomes lacking pseudouridine are deficient in internal ribosomal entry site (IRES)-dependent translational initiation due to failure of the ribosome to bind to the IRES sequence [40], [41]. In general, cap-dependent translation is inhibited at the beginning of mitosis in favor of the translation of mRNAs containing IRES elements, including one isoform of cyclin-dependent kinase 11, which is necessary for progression through M phase [42], [43]. In X-linked dyskeratosis congenita, it is thought that pseudouridine-deficient ribosomes are unable to recognize IRES elements, causing a stall at M phase due to decreased translation of cell-cycle related proteins. In the same vein, it is conceivable that changes in rRNA methylation or folding occasioned by the D86G substitution in EMG1 alter the specificity of the ribosome in such a way that it can no longer recognize or bind IRES elements efficiently. Further support for this hypothesis comes from the fact that IRES-dependent mRNA translation is down-regulated in mice expressing mutated *Rps19*, the gene most commonly associated with Diamond–Blackfan anemia [44]. It has been postulated that EMG1 promotes RPS19 binding to the nascent ribosome [21], [22]. Further study is needed to determine if defects in IRES-mediated translation lead to the delay or failure of progression through G2/M seen in BCS patient lymphoblasts.

To determine if a defect in ribosome biogenesis was causing the delay at G2/M and the concomitant reduction in cell proliferation rates, the processing of precursor 45S rRNA to its mature forms was examined using pulse-chase metabolic labeling. We detected a slight but reproducible decrease in the processing rate of 18S rRNA in both fibroblasts and lymphoblasts, evidence that a defect in ribosome biogenesis underlies Bowen–Conradi syndrome. As EMG1 has more than one proposed role in 18S rRNA processing, it is difficult to determine the exact nature of the defect. Given that EMG1 is a methyltransferase, the use of ^3^H-methyl methionine as a label makes it impossible to determine if the reduction in detectable radioactivity is the result of reduced rRNA production, or if rRNA is produced at normal levels but is under-methylated. Several lines of evidence point to the former scenario. First, yeast Emg1 bearing the equivalent mutation of the BCS protein conserves its rRNA methyltransferase activity in an in vitro methylation assay [20] despite causing a reduction in cell growth rates. Second, Emg1 mutants that are unable to bind the methyl donor SAM maintain normal ribosome biogenesis [45]. Finally, similar results were observed using inorganic phosphate as a radiolabel, which incorporates into the RNA backbone and does not affect rRNA methylation. It is therefore likely that the reduction in EMG1 causes a delay in 45S to 18S rRNA processing via its role in RPS19 recruitment or rRNA folding rather than its catalytic activity. Despite the fact that a delay in processing was observed in both fibroblasts and lymphoblasts, steady-state ratios of 60S to 40S ribosomal subunits were unaffected in both cell types, and consequently protein synthesis rates were also unchanged. This is similar to what was found in the ribosomopathy Shwachman–Diamond syndrome, where a reduction in rRNA production in patient fibroblasts was not associated with an imbalance in 60S, 40S, or 80S ribosomal subunit levels at steady state [34]. Given the transient nature of the 18S rRNA defect observed in BCS patient cells, it is unlikely that a 60S/40S subunit ratio imbalance would be detected using sucrose gradient analysis of ribosome subunits at steady state, as we did here.

Underlining the importance of the TP53 checkpoint in proper ribosome biogenesis, previous studies have shown that removal of *Trp53* can rescue the defect in certain mouse models of ribosomopathies [46], [47]. However, crossing *Trp53*^−/−^ mice with *Emg1*^+/−^ mice failed to rescue the pre-implantation growth arrest phenotype of EMG1 knockouts [32]. Taken together with our TP53 immunoblot studies which did not show stabilization of TP53 in BCS patient cells, we concluded that the EMG1 defect likely does not activate the TP53 pathway. Although the link between the TP53 pathway and ribosome biogenesis has been exploited in animal models of ribosome biogenesis, recent studies have shown variable responses to TP53 inhibition or knockout. In *Rpl38*, *Rps19*, and *Rpl11* mutant mice, the phenotype is refractory to TRP53 inhibition [44], [48]. In a zebrafish model of Shwachman–Diamond syndrome, ablation of the TP53 checkpoint in *sbds* knockdown fish ameliorates the skeletal phenotype and improves the overall health of the embryos [49]. However, the pancreas hypoplasia is not rescued, suggesting that the phenotype is only partially mediated by the TP53 pathway. Similarly, TP53 knockdown in a Diamond–Blackfan anemia model rescued the morphological abnormalities, but did not alter the erythropoietic defect [50], [51]. The selective contribution of the TP53 checkpoint to individual ribosomopathies, and even different tissues within a single ribosomopathy, is just beginning to be appreciated. Therefore, stabilization of TP53 is still a possibility in other BCS patient tissues or cell types.

The fact that lymphoblasts were affected, while fibroblasts were not, supports the hypothesis that proper levels of EMG1 are critical in rapidly proliferating cells. Slower-growing cells, such as fibroblasts, have less of a demand for ribosomes and therefore are not as strongly affected by defects in ribosome biogenesis; thus the levels of EMG1 protein in BCS patient fibroblasts is likely adequate to allow normal rates of proliferation in these cells. By extension, the rapidly proliferating cells of the embryo could be severely affected by a delay in 18S rRNA processing, resulting in the developmental delay seen in BCS patients. The different results in lymphoblasts and fibroblasts may also be a reflection of the tissue specificity which is often seen in ribosome biogenesis disorders. The preferential sensitivity of the lymphoblasts to a reduction in EMG1 levels might be expected to result in immune system defects. This has not been recorded in BCS patients, although several cases of pneumonia, attributed to uncoordinated swallowing and aspiration, were reported [1], [2], [4]. In BCS, the phenotype primarily affects the central nervous system, making it likely that EMG1 deficiency has a greater impact on the rapidly developing brain than it does on other tissues. These tissue-specific presentations are common in the ribosomopathies and indicate that studies of neural tissues may be required to demonstrate the full impact of EMG1 deficiency on ribosomal biogenesis. Taken together, these results confirm conservation of EMG1 function in yeast and human cells, and link BCS with the growing number of ribosomopathies.
